# Validation of two generic patient-reported outcome measures in patients with type 2 diabetes

**DOI:** 10.1186/1477-7525-5-47

**Published:** 2007-07-31

**Authors:** Louis S Matza, Kristina S Boye, Nicole Yurgin

**Affiliations:** 1 Center for Health Outcomes Research at United BioSource Corporation, Bethesda, MD, USA; 2 Eli Lilly & Company, Indianapolis, IN, USA

## Abstract

**Background:**

Prior to using a generic patient-reported outcome measure (PRO), the measure should be validated within the target population. The purpose of the current study was to validate two generic measures in patients with type 2 diabetes.

**Methods:**

Patients with type 2 diabetes in Scotland and England completed two generic measures: EQ-5D and Psychological General Well-Being Index (PGWB). Two diabetes-specific measures were administered: ADS and DSC-R. Analyses assessed reliability and validity.

**Results:**

There were 130 participants (53 Scotland; 77 England; 64% male; mean age = 55.7 years). Responses on the EQ-5D and PGWB reflected moderate impairment consistent with previous diabetes samples: mean EQ-5D Index score, 0.75; EQ-5D VAS, 68.8; PGWB global score, 67.9. All scales of the PGWB demonstrated good internal consistency reliability (Cronbach's alpha = 0.77 to 0.97). The EQ-5D and PGWB demonstrated convergent validity through significant correlations with the ADS (r = 0.48 to 0.61), DSC-R scales (r = 0.33 to 0.81 except ophthalmology subscale), and Body Mass Index (r = 0.15 to 0.38). The EQ-5D and PGWB discriminated between groups of patients known to differ in diabetes-related characteristics (e.g., history of hypoglycemia).

**Conclusion:**

Results support the use of the EQ-5D and PGWB among patients with type 2 diabetes, possibly in combination with condition-specific measures.

## Background

Patient-reported outcome measures (PROs) that assess health-related quality of life (HRQL) and related constructs are often categorized as either generic or condition-specific [1-3]. Generic measures are designed for use among diverse populations with a broad range of medical conditions. These instruments can also be used to characterize healthy samples without a particular medical condition. In contrast, condition-specific measures are relevant to a particular group of patients, and they have been developed to assess specific populations, quantify aspects of functioning, and examine the impact of particular medical conditions or treatments.

A substantial body of literature has focused on comparing generic and condition-specific measures, while identifying advantages of each. Compared with generic measures, the primary advantage of condition-specific measures is that they are usually found to be more responsive to treatment-related change [4-6]. Because of their greater responsiveness to change, condition-specific instruments may be more likely to detect differences between treatment groups in clinical trials [7]. An advantage of generic PROs is that they can be used to compare among various populations, make comparisons to the general population, and estimate the relative impact of various medical conditions or treatments [1,2,8,9]. Generic measures also tend to correlate well with condition-specific measures [10,11], and in some studies, they have demonstrated responsiveness or convergent validity that was comparable to condition-specific measures [12-16]. Most importantly, generic measures are distinct from condition-specific measures in that they usually assess impact of disease and treatment on overall functioning or a broader range of health domains [8,17].

Because generic and condition-specific measures have different strengths and are conceptually distinct, it is often recommended to administer both types of instruments as part of a complete outcomes assessment in clinical trials [5,17-19]. Prior to using a generic measure, however, it is important that the instrument is shown to be reliable and valid in the specific population under investigation [9]. Instruments validated in one population will not necessarily perform well among patients with a different medical condition. Therefore, it is often necessary to validate generic PROs in multiple populations. The purpose of the current study was to examine the performance of two commonly used generic PROs in a sample of patients with type 2 diabetes, an increasingly prevalent disease associated with serious health risks and HRQL impairment [20-27].

One of the two measures evaluated in this study is the EuroQol EQ-5D, a brief health status instrument frequently used for clinical and economic appraisal [28,29]. The EQ-5D has been used in several large studies involving patients with type 2 diabetes to provide an estimate of HRQL and derive utilities, which used to compute quality-adjusted life years (QALYs) in models evaluating treatment cost-effectiveness [30-35]. However, no studies were located focusing on validation of the EQ-5D among patients with type 2 diabetes. The other generic PRO examined in this study is the Psychological General Well-Being Index (PGWB), which was developed to measure self-perception of affective states and to assess a sense of subjective well-being or distress [36]. The PGWB has been used in several studies that included patients with type 2 diabetes [37,38], including one study that found adequate internal consistency reliability and convergent validity among a sample of 88 Native American patients with type 2 diabetes [39]. However, no studies were located validating the PGWB in a more general sample of patients with type 2 diabetes. The current analysis builds on these findings by evaluating these properties among a larger sample in the United Kingdom, while also assessing known-groups validity.

## Methods

### Participants

Participants were required to be (1) diagnosed with type 2 diabetes by a recognized medical professional (as indicated by patient self-report); (2) between 30 and 75 years old; (3) able to identify the age at which they were first diagnosed with diabetes; (4) able to read and understand English; and (5) willing and able to give informed consent prior to study entry. Patients were not eligible if they had cognitive impairment, hearing difficulty, or severe psychopathology that could interfere with the ability to complete the study measures. Participants were recruited through ten advertisements placed in four newspapers in Scotland and England from June to August of 2005. Interested patients responded by telephone, and 200 potential participants were screened to assess whether they met the study inclusion/exclusion criteria. Of the 200 screened patients, 132 were available, eligible, and willing to participate. Two participants who attended the interview were unable to complete the questionnaires relevant to the current analysis. Thus, the current study includes a sample of 130 individuals with type 2 diabetes.

### Measures

#### EuroQol EQ-5D

This questionnaire assesses functioning in five dimensions: mobility, self-care, usual activities, pain/discomfort, and anxiety/depression. Each dimension is assessed by one item with three response options: no problems, some problems, and severe problems. Higher scores on these items indicate greater impairment. Responses to these five items are used to derive the weighted EQ-5D index score, which represents overall health with a possible range from -0.594 to 1.0 [40,41]. After completing the five dimension items, patients completed the single item EQ-5D visual analogue scale (VAS), on which they rated their current health on a scale ranging from 0 (worst imaginable health state) to 100 (best imaginable health state). Higher scores on the index score and VAS indicate better health status.

#### Psychological General Well-Being Index (PGWB)

The PGWB is comprised of 22 items in the following 6 dimensions: anxiety (5 items); depressed mood (3 items); positive well-being (4 items); self-control (3 items); general health (3 items); and vitality (4 items). Responses are rated on a six-point Likert scale ranging from 0 (reflecting the most distress) to 5 (reflecting the highest level of well-being) [42]. Scoring approaches for the PGWB have varied in previously published studies, with the global score often ranging from 0 to 100 (when items range from 0 to 5) or 22 to 132 (when items range from 1 to 6). For the current study, this instrument was scored following the approach proposed by Chassany et al. [42] in the recently published user's manual. The six PGWB raw subscale scores were computed by summing the item responses, and the raw global score is the sum of the six subscale scores. Then, these raw scores were transformed to a 0–100 scale by dividing each raw score by the maximum possible score, multiplying by 100, and rounding to one decimal place. Higher scores reflect better well-being.

#### Appraisal of Diabetes Scale (ADS)

The ADS is a brief 7-item patient-reported scale that assesses the impact of diabetes on a patient's life. This condition-specific instrument was used to assess criterion validity of the EQ-5D and PGWB. The items, which were developed based on theory and previous research, include "How upsetting is having diabetes for you?" and "To what degree does your diabetes get in the way of your developing life goals?". The instrument has been shown to have acceptable internal consistency reliability, test-retest reliability, and construct validity [43]. Higher scores indicate greater negative impact of diabetes.

#### Diabetes Symptom Checklist – Revised (DSC-R)

The DSC-R is a revised version of the DSC-2, which was developed to measure both the frequency and perceived discomfort of physical and psychological symptoms associated with type 2 diabetes and its potential complications [44]. Like the ADS, the DSC-R was used to assess validity of the two generic instruments that were the focus of the current analysis. For each of the 34 DSC-R items, respondents first indicate whether they have experienced each symptom in the past month by circling "yes" or "no." If the patient answers "no," then the item is scored as a 0. If "yes" is selected, the participant proceeds to rate the perceived discomfort of the symptom on a 5-point scale ranging from 1 (not at all) to 5 (extremely). The instrument yields a total score and scores on the following subscales: psychology-fatigue, psychology-cognitive, neurology-pain, neurology-sensory, cardiology, ophthalmology, hypoglycemia, and hyperglycemia. The total score and all subscale scores range from 0 to 5, with higher scores indicating greater symptom burden.

### Demographic and clinical information form

Patients completed a brief questionnaire that included questions on age, sex, ethnicity, living situation, employment, education, diabetes-related health, and general health. Participants' weight and height were measured at the beginning of each interview and recorded on the demographic and clinical information form so that patients' Body Mass Index (BMI) could be computed [45]. Items assessing diabetes-related health included questions asking whether participants had ever experienced hyperglycemia, daytime hypoglycemia, or nighttime hypoglycemia.

### Data collection and statistical analysis procedures

Data were collected in Edinburgh and London during August 2005. All procedures and instruments were approved by an independent Institutional Review Board, and all participants provided written informed consent prior to completing any study measures. After signing the consent form, participants independently completed the questionnaires analyzed in the current study.

Statistical analyses were completed using SAS version 8.12 (SAS Institute, Cary, NC). Descriptive statistics were used to summarize demographic/clinical characteristics and scores on health status measures. Categorical variables are summarized in terms of frequencies and percentages, and for each continuous variable, the mean, standard deviation, median, range, percent at floor, and percent and ceiling are presented. In the current study, there were no missing data on any of the measures, and therefore, procedures for handling missing data were not followed. Internal consistency reliability is the extent to which individual items within a scale are related to one another. Internal consistency was examined for the PGWB subscales and global score using Cronbach's formula for coefficient alpha. Cronbach's alphas greater than 0.70 are generally considered acceptable [9].

Construct validity refers to the extent to which the instrument measures what it is intended to measure. To assess the construct validity of the EQ-5D and PGWB, both convergent and known-groups validity were examined. Convergent validity is the degree to which scores from the instrument undergoing evaluation are related to scores from an instrument measuring a similar construct. To examine convergent validity, Spearman correlations were performed to examine the relationship of the two generic measures with diabetes-specific patient-reported measures and patients' BMI. BMI was used as a criterion because body weight is an important health indicator for patients with type 2 diabetes. Roughly 40% to 50% of patients with diabetes meet criteria for obesity [26,46], and obesity is likely to exacerbate symptoms and metabolic abnormalities of type 2 diabetes, increase the risk of complications, and complicate the goal of achieving glycemic control [47-50]. Correlation coefficients were interpreted based on guidelines proposed by Cohen [51] suggesting that a correlation of 0.10 is small, 0.30 is moderate, and 0.50 is large. It was hypothesized that the generic measures under investigation would be significantly correlated with the condition-specific measures and patients' BMI, with correlation coefficients in the moderate to large range.

Known-groups validity is a scale's ability to discriminate among groups of patients who are known to differ by a key indicator. Using t-tests, EQ-5D and PGWB scores were compared among groups of patients who differed in the following characteristics: symptom burden as reported on condition-specific measures; type of pharmacological treatment (injectable insulin vs. oral medication only); preference for weight change (patients who would like to lose weight vs. patients who would like to stay the same weight); and experience with daytime hypoglycemia, nighttime hypoglycemia, and hyperglycemia (patients who had each of these experiences vs. patients who did not). It was hypothesized that the EQ-5D and PGWB would discriminate between groups of patients in each of these comparisons.

## Results

### Sample description

A total of 130 eligible participants completed the study, 53 in Edinburgh and 77 in London (Table 1). The sample in London was more ethnically diverse than the sample in Edinburgh, which was 100% White. There were no other statistically significant differences between the two geographic groups with respect to demographics (e.g., gender, age, marital status, employment) or clinical characteristics (e.g., BMI, age when first diagnosed with diabetes, current treatment). Therefore, data from the two cities were pooled for all analyses.

**Table 1:** Demographic and clinical characteristics

**Characteristic**	**Total Sample (N = 130)**	**Edinburgh (N = 53)**	**London (N = 77)**	**p value^1^**
**Age (mean, SD)**	55.7 (10.3)	56.2 (8.8)	55.4 (11.3)	0.69
**Gender (n, %)**				
Male	84 (64.6%)	33 (62.3%)	51 (66.2%)	0.71
Female	46 (35.4%)	20 (37.7%)	26 (33.8%)	
**Ethnicity (n, %)**				
White	105 (80.8%)	53 (100.0%)	52 (67.5%)	<0.0001
Black	8 (6.2%)	0 (0.0%)	8 (10.4%)	
Indian	8 (6.2%)	0 (0.0%)	8 (10.4%)	
Other	9 (6.9%)	0 (0.0%)	9 (11.7%)	
**Marital Status (n, %)**				
Married	79 (60.8%)	37 (69.8%)	42 (54.5%)	0.10
Not married	51 (39.2%)	16 (30.2%)	35 (45.5%)	
**Employment Status (n, %)**				
Full-time work	46 (35.4%)	19 (35.8%)	27 (35.1%)	0.91
Retired	47 (36.2%)	20 (37.7%)	27 (35.1%)	
Other	37 (28.5%)	14 (26.4%)	23 (29.9%)	
**Education Level (n, %)**				
Did not complete college degree	86 (66.2%)	40 (75.5%)	46 (59.7%)	0.089
Completed college degree or higher	44 (33.8%)	13 (24.5%)	31 (40.3%)	
**Body Mass Index^2^**	31.5 (5.4)	32.3 (5.6)	31.0 (5.3)	0.19
**Age when Diagnosed with Diabetes (mean, SD)**	49.1 (11.2)	49.1 (11.2)	49.2 (11.3)	0.96
**Current Treatment (n, %)**				
Diet and/or exercise alone	14 (10.8%)	6 (11.3%)	8 (10.4%)	0.34
Oral meds alone (no injectable)	85 (65.4%)	31 (58.5%)	54 (70.1%)	
Injectable medication with or without other treatment	31 (23.8%)	16 (30.2%)	15 (19.5%)	
**Other Health Conditions (n, %)**				
Hypertension (n, %)	48 (36.9%)	17 (32.1%)	31 (40.3%)	0.36
Diabetic retinopathy (n, %)	7 (5.4%)	3 (5.7%)	4 (5.2%)	1.00
Depression or other mental health condition (n, %)	17 (13.1%)	7 (13.2%)	10 (13.0%)	1.00
Other Health Condition(s) (n, %)	56 (43.1%)	25 (47.2%)	31 (40.3%)	0.47
None (n, %)	45 (34.6%)	16 (30.2%)	29 (37.7%)	0.45

^1 ^P value is for comparisons between the Edinburgh sample and the London sample. Continuous variables compared with t-tests; 2-level categorical variables such as gender compared with Fisher's exact test; categorical variables with more than two levels were compared with chi-squares.

^2 ^Body Mass Index = (weight in pounds/[height in inches]^2^) × 703

The majority of participants were male (n = 84; 64.6%), and the mean age of the total sample was 55.7 years old. Most of the patients were currently married (n = 79; 60.8%), over a third worked full-time (n = 46; 35.4%), and over a third were retired (n = 47; 36.2%). The mean Body Mass Index (BMI) of the total sample was 31.5, which is considered to be in the obese range [45]. The mean age when first diagnosed with diabetes was 49.1 years old. Most participants received oral, but not injectable medications for treatment of their diabetes (n = 85; 65.4%). A total of 31 participants (23.8%) were treated with injectable medications, either alone or in combination with oral medications.

### Descriptive statistics and internal consistency reliability

There were no missing data on the EQ-5D (Table 2). The great majority of the 130 participants reported no problems on the EQ-5D self-care item (n = 120; 92.3%), while the other four dimension items reflected greater rates of difficulty. Roughly one third of participants reported having at least some problems in the dimensions of mobility (n = 42; 32.3%) and usual activities (n = 41; 31.5%). Approximately a third of patients reported having either some problems (n = 35; 26.9%) or extreme problems (n = 8; 6.2%) in the anxiety/depression dimension. The greatest rates of difficulty were found in the pain/discomfort dimension, with half of the sample reporting either some problems (n = 52; 40.0%) or extreme problems (n = 13; 10.0%). The pattern of responses was similar in the London and Edinburgh samples, although the participants in Edinburgh reported slightly greater rates of problems in mobility, self-care, and usual activities. The mean EQ-5D index score of 0.75 and VAS score of 68.8 both indicate a moderate level of overall impairment in this sample (Table 3). The mean index score was somewhat lower for the Edinburgh sample (0.70) than for the London sample (0.79). Analysis of floor and ceiling effects revealed that 40% of the sample had the maximum EQ-5D index score of 1. The VAS did not have a similar ceiling effect.

**Table 2:** Frequencies and percentages of participants' responses to the five categorical items of the EQ-5D

**Responses**	**EQ-5D Items**				
	**Mobility**	**Self-Care**	**Usual Activities**	**Pain/Discomfort**	**Anxiety/Depression**
**Edinburgh Sample (n = 53)**					
No problems	34 (64.2%)	47 (88.7%)	33 (62.3%)	26 (49.1%)	38 (71.7%)
Some problems	19 (35.9%)	6 (11.3%)	19 (35.9%)	19 (35.9%)	9 (17.0%)
Extreme problems	0 (0.0%)	0 (0.0%)	1 (1.9%)	8 (15.1%)	6 (11.3%)
**London Sample (n = 77)**					
No problems	54 (70.1%)	73 (94.8%)	56 (72.7%)	39 (50.7%)	49 (63.6%)
Some problems	23 (29.9%)	4 (5.2%)	21 (27.3%)	33 (42.9%)	26 (33.8%)
Extreme problems	0 (0.0%)	0 (0.0%)	0 (0.0%)	5 (6.5%)	2 (2.6%)
**Total Sample (n = 130)**					
No problems	88 (67.7%)	120 (92.3%)	89 (68.5%)	65 (50.0%)	87 (66.9%)
Some problems	42 (32.3%)	10 (7.7%)	40 (30.8%)	52 (40.0%)	35 (26.9%)
Extreme problems	0 (0.0%)	0 (0.0%)	1 (0.8%)	13 (10.0%)	8 (6.2%)

**Table 3:** Distributional Characteristics of EQ-5D Continuous Scales

**EQ-5D Scales**	**Mean**	**SD**	**Range**	**% at Floor**	**% at Ceiling**
**Edinburgh Sample (n = 53)**					
Index Score	0.70	0.36	-0.24–1.0	0%	41.5%
VAS	68.4	19.1	10.0–98.0	0%	0%
**London Sample (n = 77)**					
Index Score	0.79	0.25	-0.18–1.0	0%	39.0%
VAS	69.1	17.9	11.0–95.0	0%	0%
**Total Sample (n = 130)**					
Index Score	0.75	0.30	-0.24–1.0	0%	40.0%
VAS	68.8	18.3	10.0–98.0	0%	0%

VAS = Visual Analog Scale

On the PGWB, there were no missing data, and mean subscale scores were similar in the two samples (Table 4). The greatest impairment was reflected in the vitality subscale, which had a mean score of 59.5 in the total sample of 130 participants. The positive well-being and general health subscales also reflected some impairment (mean scores of 61.1 and 63.4, respectively). Higher scores were found on the anxiety, depressed mood, and self-control subscales (69.9, 78.9, and 78.0, respectively). On the depressed mood subscale, 23.8% of the sample had the maximum score of 100, indicating that almost a quarter of this sample reported no problems with depression. The PGWB demonstrated good internal consistency reliability, with Cronbach's alphas for the six subscales ranging from 0.77 to 0.92 (Table 5). The alpha for the global score was 0.97.

**Table 4:** Distributional Characteristics of the PGWB Scales

**PGWB Scales**	**Mean**	**SD**	**Range**	**% at Floor**	**% at Ceiling**
**Edinburgh Sample (n = 53)**					
Anxiety	70.5	23.9	0.0–100.0	1.9%	9.4%
Depressed Mood	78.0	23.9	13.3–100.0	0%	22.6%
Positive Well-Being	59.9	22.5	0.0–95.0	1.9%	0%
Self-Control	76.5	23.0	0.0–100.0	1.9%	17.0%
General Health	62.3	22.6	0.0–100.0	1.9%	1.9%
Vitality	57.3	22.1	0.0–100.0	1.9%	1.9%
Global Score	66.9	20.9	3.6–94.5	0%	0%
**London Sample (n = 77)**					
Anxiety	69.5	20.7	12.0–100.0	0%	3.9%
Depressed Mood	79.6	20.9	26.7–100.0	0%	24.7%
Positive Well-Being	61.9	21.9	5.0–100.0	0%	1.3%
Self-Control	79.0	18.8	26.7–100.0	0%	15.6%
General Health	64.2	19.3	20.0–100.0	0%	2.6%
Vitality	61.1	21.3	0.0–100.0	1.3%	1.3%
Global Score	68.5	18.1	16.4–94.5	0%	0%
**Total Sample (n = 130)**					
Anxiety	69.9	22.0	0.0–100.0	0.8%	6.2%
Depressed Mood	78.9	22.1	13.3–100.0	0%	23.8%
Positive Well-Being	61.1	22.1	0.0–100.0	0.8%	0.8%
Self-Control	78.0	20.6	0.0–100.0	0.8%	16.2%
General Health	63.4	20.6	0.0–100.0	0.8%	2.3%
Vitality	59.5	21.6	0.0–100.0	1.5%	1.5%
Global Score	67.9	19.2	3.6–94.5	0%	0%

PGWB = Psychological General Well-Being Index

**Table 5:** Internal consistency reliability of the PGWB

**PGWB**	**Number of items (k)**	**Cronbach's alpha (std)**
Anxiety	5	0.92
Depressed Mood	3	0.88
Positive Well-Being	4	0.90
Self-Control	3	0.80
General Health	3	0.77
Vitality	4	0.90
PGWB Global Score	22	0.97

PGWB = Psychological General Well-Being Index

### Convergent validity

Convergent validity of the EQ-5D index score and VAS were demonstrated through significant correlations with the ADS, DSC-R, and patients' BMI (Table 6). These correlations were generally in the moderate to large range. Spearman correlations of the index score and VAS with the ADS had coefficients of -0.52 and -0.49, respectively (both p < 0.001). Correlations with the DSC-R total score were -0.64 and -0.53 (both p < 0.001). Correlations between the EQ-5D and the DSC-R subscales ranged from -0.33 to -0.61 (all p < 0.001), except for the correlations with the ophthalmology subscale which were somewhat smaller (r = -0.22 and -0.19; both p < 0.05). Correlations of the EQ-5D index score and VAS with patients' BMI were -0.27 (p < 0.01) and -0.38 (p < 0.001).

**Table 6:** Convergent Validity of the EQ-5D and PGWB: Spearman correlations with condition-specific measures and BMI

			**DSC-R Scales**								
	**ADS**	**Total Score**	Fatigue	Cognitive	Pain	Sensory	Cardiology	Ophthalmology	Hypoglycemia	Hyperglycemia	**BMI^1^**
**EQ-5D**											
Index Score	-.52***	-.64***	-.61***	-.46***	-.51***	-.53***	-.56***	-.22*	-.44***	-.46***	-.27**
VAS	-.49***	-.53***	-.50***	-.43***	-.39***	-.41***	-.45***	-.19*	-.33***	-.37***	-.38***
**PGWB**											
Global Score	-.61***	-.71***	-.76***	-.61***	-.46***	-.48***	-.54***	-.24**	-.56***	-.45***	-.24**
Anxiety	-.48***	-.58***	-.62***	-.50***	-.38***	-.37***	-.43***	-.19*	-.52***	-.37***	-.15
Depressed Mood	-.55***	-.57***	-.65***	-.52***	-.37***	-.33***	-.48***	-.15	-.48***	-.34***	-.16
Positive Well-Being	-.61***	-.60***	-.65***	-.56***	-.39***	-.40***	-.45***	-.19*	-.52***	-.34***	-.19*
Self-Control	-.54***	-.61***	-.61***	-.60***	-.40***	-.42***	-.47***	-.25**	-.49***	-.38***	-.20*
General Health	-.52***	-.68***	-.66***	-.47***	-.50***	-.58***	-.50***	-.32***	-.44***	-.45***	-.28**
Vitality	-.58***	-.67***	-.81***	-.59***	-.38***	-.45***	-.49***	-.19*	-.50***	-.43***	-.28***

BMI = Body Mass Index; ADS = Appraisal of Diabetes Scale; DSC-R = Diabetes Symptom Checklist – Revised; PGWB = Psychological General Well-Being Index; VAS = Visual Analog Scale; ^1^Body Mass Index = (weight in pounds/[height in inches]^2^) × 703; *p < 0.05; **p < 0.01; ***p < 0.001

Convergent validity of the PGWB global score was also supported (Table 6), with the majority of correlations in the moderate to large range. Correlations of the PGWB global score with the ADS and the DSC-R total score had coefficients of -0.61 and -0.71, respectively (both p < 0.001). Correlations of the PGWB global score with the DSC-R subscales, except the ophthalmology subscale, ranged from -0.45 to -0.76 (all p < 0.001). The correlation with patients' BMI was somewhat smaller, but still statistically significant (r = -0.24; p < 0.01).

The PGWB subscales also demonstrated good convergent validity. Correlations of these six subscales with the ADS ranged from -0.48 to -0.61 (all p < 0.001). Similarly, all PGWB subscales had correlations with the DSC-R total score that were in the large range (r = -0.57 to -0.68; all p < 0.001). Correlations between PGWB subscales and DSC-R subscales were all in the moderate to large range and statistically significant (p < 0.001), except for those involving the DSC-R ophthalmology subscale which were notably smaller. Correlations with BMI were statistically significant for four PGWB subscales: positive well-being (r = -0.19), self-control (r = -0.20), general health (r = 0.28), and vitality (r = 0.28). The anxiety and depressed mood subscales of the PGWB were not significantly correlated with BMI.

### Known-groups validity

Known-groups validity of the EQ-5D index score and VAS was supported in several group comparisons (Table 7). Both EQ-5D scores significantly discriminated between groups of patients categorized based on median splits of their ADS score and DSC-R total score (all p < 0.001). Groups with higher scores (indicating greater symptom burden) on these two diabetes-specific instruments had lower EQ-5D scores (indicating lower HRQL). In addition, patients who wanted to lose weight (n = 113) had significantly lower EQ-5D scores than patients who wanted to stay the same weight (n = 16) (both p < 0.001). Patients who had experienced daytime hypoglycemia had significantly lower mean EQ-5D index (p < 0.05) and VAS (p < 0.001) scores than patients who had not experienced daytime hypoglycemia. Results followed a similar pattern for hyperglycemia and nighttime hypoglycemia, although comparisons were not consistently statistically significant.

**Table 7:** Known-groups validity of the EQ-5D and PGWB Global Score: Comparisons between groups of patients

**Variable Used to Differentiate Groups**	**Groups**	**EQ-5D Index Score**		**EQ-5D VAS**		**PGWB Global Score**	
		**Mean**	**t Value**	**Mean**	**t Value**	**Mean**	**t Value**
ADS Median Split	ADS Score ≤ 16 (n = 58)	0.88	5.1***	76.7	5.0***	78.9	7.3***
	ADS Score > 16 (n = 72)	0.65		62.5		59.0	
DSC-R Median Split	DSC-R Total Score ≤ 0.7 (n = 61)	0.91	6.6***	78.8	7.0***	79.6	8.2***
	DSC-R Total Score > 0.7 (n = 69)	0.61		60.0		57.5	
Preference for Weight Change	Lose weight (n = 113)	0.73	-4.4***	66.7	-5.0***	66.5	-2.0*
	Stay same (n = 16)	0.92		83.0		76.9	
Experienced Hypoglycemia During the Day	Yes (n = 53)	0.68	-2.6*	62.6	-3.4***	64.0	-2.3*
	No (n = 74)	0.82		73.9		71.8	
Experienced Hypoglycemia During the Night	Yes (n = 23)	0.60	-3.2**	64.2	-1.5	58.8	-3.0**
	No (n = 101)	0.80		70.6		71.0	
Experienced Hyperglycemia	Yes (n = 64)	0.73	-1.1	64.5	-2.8**	64.9	-2.1*
	No (n = 63)	0.78		73.4		71.6	
Type of Treatment	Injectable (n = 31)	0.65	-1.8	61.4	-2.0	62.6	-1.3
	Oral only (n = 85)	0.78		70.2		68.9	

ADS = Appraisal of Diabetes Scale; DSC-R = Diabetes Symptom Checklist – Revised; PGWB = Psychological General Well-Being Index; VAS = Visual Analog Scale; *p < 0.05; **p < 0.01; ***p < 0.001

The PGWB also discriminated between groups of patients who differed in scores on diabetes-specific instruments and clinical characteristics (Table 7). Groups with higher scores (indicating greater symptom burden) on the ADS and DSC-R total score had lower PGWB global scores (indicating lower overall well-being) (both p < 0.001). Patients who wanted to lose weight (n = 113) had a significantly lower mean PGWB global score than patients who wanted to stay the same weight (n = 16) (p < 0.05). The PGWB global score also discriminated between groups of patients differing with regard to whether they had experienced daytime hypoglycemia, nighttime hypoglycemia, and hyperglycemia (all p < 0.05). Known-groups validity of the PGWB subscales was also supported (Table 8). Statistically significant group differences were found in most comparisons involving the anxiety, positive well-being, self-control, general health, and vitality subscales. The depressed mood subscale discriminated between groups of patients determined by median splits on the ADS and DSC-R, but significant differences on this subscale were not found in the other group comparisons.

**Table 8:** Known-Groups Validity of the PGWB Subscales: Comparisons between Groups of Patients

**Variable Used** ** to Differentiate Groups**	**Groups**	**Anxiety**		**Depressed ** **Mood**		**Positive** **Well Being**		**Self Control**		**General Health**		**Vitality**	
		Mean	t Value	Mean	t Value	Mean	t Value	Mean	t Value	Mean	t Value	Mean	t Value
ADS Median Split	ADS Score ≤ 16 (n = 58)	79.9	5.4***	89.7	5.9***	74.3	7.6***	88.4	6.1***	73.1	5.5***	71.2	6.5***
	ADS Score > 16 (n = 72)	61.8		70.3		50.4		69.6		55.7		50.1	
DSC-R Median Split	DSC-R Total Score ≤ 0.7 (n = 61)	81.0	6.3***	89.5	5.9***	72.5	6.3***	89.0	6.8***	76.1	8.1***	73.0	8.4***
	DSC-R Total Score > 0.7 (n = 69)	60.0		69.6		50.9		68.3		52.3		47.6	
Preference for Weight Change	Lose weight (n = 113)	68.5	-1.9	77.9	-1.4	60.2	-1.2	77.1	-1.3	61.2	-3.1**	57.9	-2.1*
	Stay same (n = 16)	79.5		86.3		67.5		84.2		77.9		70.0	
Experienced Hypoglycemia During the Day	Yes (n = 53)	65.7	-2.2*	75.8	-1.5	57.1	-2.1*	74.2	-2.1*	58.7	-2.5*	56.0	-2.0*
	No (n = 74)	73.9		82.1		65.1		82.0		67.7		63.4	
Experienced Hypoglycemia During the Night	Yes (n = 23)	60.5	-2.6**	70.4	-2.0	50.0	-3.1**	66.1	-3.7***	55.7	-2.2*	53.5	-1.9
	No (n = 101)	73.0		82.1		64.8		81.8		65.8		62.3	
Experienced Hyperglycemia	Yes (n = 64)	67.3	-1.5	76.3	-1.6	57.5	-2.0*	75.2	-1.9	62.4	-0.7	54.8	-2.9**
	No (n = 63)	73.1		82.3		65.2		81.7		65.0		65.2	
Type of Treatment	Injectable (n = 31)	65.5	-1.1	73.5	-1.2	54.7	-1.6	74.8	-0.7	58.7	-1.4	52.4	-1.6
	Oral only (n = 85)	70.9		80.1		62.5		78.0		64.7		61.0	

ADS = Appraisal of Diabetes Scale; DSC-R = Diabetes Symptom Checklist – Revised; PGWB = Psychological General Well-Being Index; VAS = Visual Analog Scale; *p < 0.05; **p < 0.01; ***p < 0.001

No scales of the EQ-5D or PGWB significantly discriminated between patients treated with injectable medication (i.e., insulin with or without concomitant oral medication; n = 31) and patients treated only with oral medications. In general, scores were somewhat higher in the oral treatment group, but these differences between groups were not statistically significant.

## Discussion

Current findings provide support for the use of the EQ-5D and PGWB in patients with type 2 diabetes. The PGWB had good internal consistency reliability, and both instruments demonstrated excellent convergent and known-groups validity. Convergent validity was supported through consistently significant correlations with self-report, diabetes-specific symptom impact measures as well as patients' BMI, an objective health indicator that is particularly relevant to type 2 diabetes [20,26,52]. Furthermore, both the EQ-5D and PGWB discriminated between groups of patients who differed in self-reported impact of diabetes symptoms, preference for weight change, and experience with hyperglycemia and hypoglycemia.

The mean EQ-5D index score of 0.75 is consistent with EQ-5D ratings in other studies of patients with type 2 diabetes. For example, the mean index score was 0.74 in a sample of 1348 patients in the Netherlands [34] and 0.76 in a sample of 4189 patients from five European countries [32]. In previous studies, the PGWB has been scored in several ways (as described in the methods section of the current manuscript), thus making it difficult to compare among studies. In the current study, this instrument was scored using a relatively new standardized approach proposed in the recently published PGWB user's manual. Using this approach, all subscale scores and the global score are normalized so that scores have a possible range of 0 to 100 [42]. The current results provide a benchmark for these normalized scores among a general sample of patients with type 2 diabetes in the UK.

Although the two generic measures were found to be valid in this sample, it would not be ideal to use either instrument as the only PRO in a clinical trial. A published analysis comparing generic to condition-specific measures across 43 randomized clinical trials found that the condition-specific measures were more responsive to change, particularly in studies with a nonzero therapeutic effect [6]. Thus, interpretation of trial outcomes based solely on generic instruments such as the PGWB or EQ-5D could fail to detect true treatment-related improvement, even though these two measures do appear to correlate well with condition-specific instruments. Furthermore, generic instruments may not capture the specific impairments within a particular population. For example, although all participants in the current sample had type 2 diabetes, 40% of the participants had the maximum EQ-5D index score of 1 which theoretically represents perfect health status. This ceiling effect suggests that the brief EQ-5D may not reflect the health-related problems of all patients with type 2 diabetes, particularly patients whose symptoms have an impact on functional domains other than the five EQ-5D dimensions. Given the different strengths of generic and condition-specific measures, we recommend using the EQ-5D and PGWB as part of an overall health outcomes battery that also includes condition-specific measures of symptom burden or HRQL. There are several well-validated instruments designed specifically to assess outcomes of treatment for type 2 diabetes [53,54], and the decision regarding which measures to use will depend on the specific aims of each study. The generic measures examined in the current study can complement previously validated condition-specific measures by providing an estimate of overall HRQL and allowing for comparisons across trials and populations.

Results of the current study are limited by the fact that data are only available at one point in time. Consequently, neither test-retest reliability nor responsiveness to change could be evaluated. Evaluation of responsiveness using longitudinal data is needed to ensure that the EQ-5D and PGWB would be sensitive to change in patients' condition when true change has occurred in either a clinical trial or naturalistic setting. It is hoped that longitudinal studies will build on the current findings by examining these measurement properties of the EQ-5D, PGWB, and other generic instruments in patients with type 2 diabetes.

## Conclusion

Psychometric instrument evaluation is an ongoing process, and confidence in a PRO's performance is strengthened as data accumulate from multiple studies and samples [55]. The current study provides initial data suggesting that the EQ-5D and PGWB are appropriate for use in patients with type 2 diabetes, and future research may provide additional support for this conclusion.

## Abbreviations

Appraisal of Diabetes Scale: ADS

Body Mass Index: BMI

Diabetes Symptom Checklist – Revised: DSC-R

health-related quality of life: HRQL

Patient-reported outcome measures: PROs

Psychological General Well-Being Index: PGWB

## Competing interests

LM works for UBC, a company that received funds from Eli Lilly for this research. KB is an employee and stock holder of Eli Lilly and Company. NY is an employee and stock holder of Eli Lilly and Company.

## Authors' contributions

LM wrote the manuscript and guided data collection, analysis, and interpretation. KS assisted with study design, provided input into data analyses/interpretation, and critically reviewed the manuscript. NY assisted with study design, provided input into data analyses/interpretation, and critically reviewed the manuscript. All authors have read and approved the final manuscript.
